# Supplementary material for: Structural basis for recognition of antihistamine drug by human histamine receptor
Source: Nat Commun. 2022 Oct 15;13:6105. doi: 10.1038/s41467-022-33880-y (PMC9569329; doi:10.1038/s41467-022-33880-y)
Supplement: Supplementary file 1 — Supplementary Information [file 41467_2022_33880_MOESM1_ESM.pdf]

Supplementary Information

Structural basis for recognition of antihistamine drug by human histamine receptor

Xueqian Peng[1], Linlin Yang[2], Zixuan Liu[1], Siyi Lou[1], Shiliu Mei[1], Meiling Li[2], Zhong Chen[3], Haitao Zhang[1,4*]

[1]Hangzhou Institute of Innovative Medicine, Institute of Pharmacology and Toxicology, Zhejiang Province Key Laboratory of Anti-Cancer Drug Research, College of Pharmaceutical Sciences, Zhejiang University, Hangzhou 310058, Zhejiang, China

[2]Department of Pharmacology, School of Basic Medical Sciences, Zhengzhou University, Zhengzhou 450052, Henan, China

[3]Key Laboratory of Neuropharmacology and Translational Medicine of Zhejiang Province, College of Pharmaceutical Sciences, Zhejiang Chinese Medical University, Hangzhou 310053, Zhejiang, China

[4]The Second Affiliated Hospital, Zhejiang University School of Medicine, Hangzhou 310009, Zhejiang, China

*Correspondence: haitaozhang@zju.edu.cn

Supplementary Figures 1-7

Supplementary Tables 1-5

Supplementary References

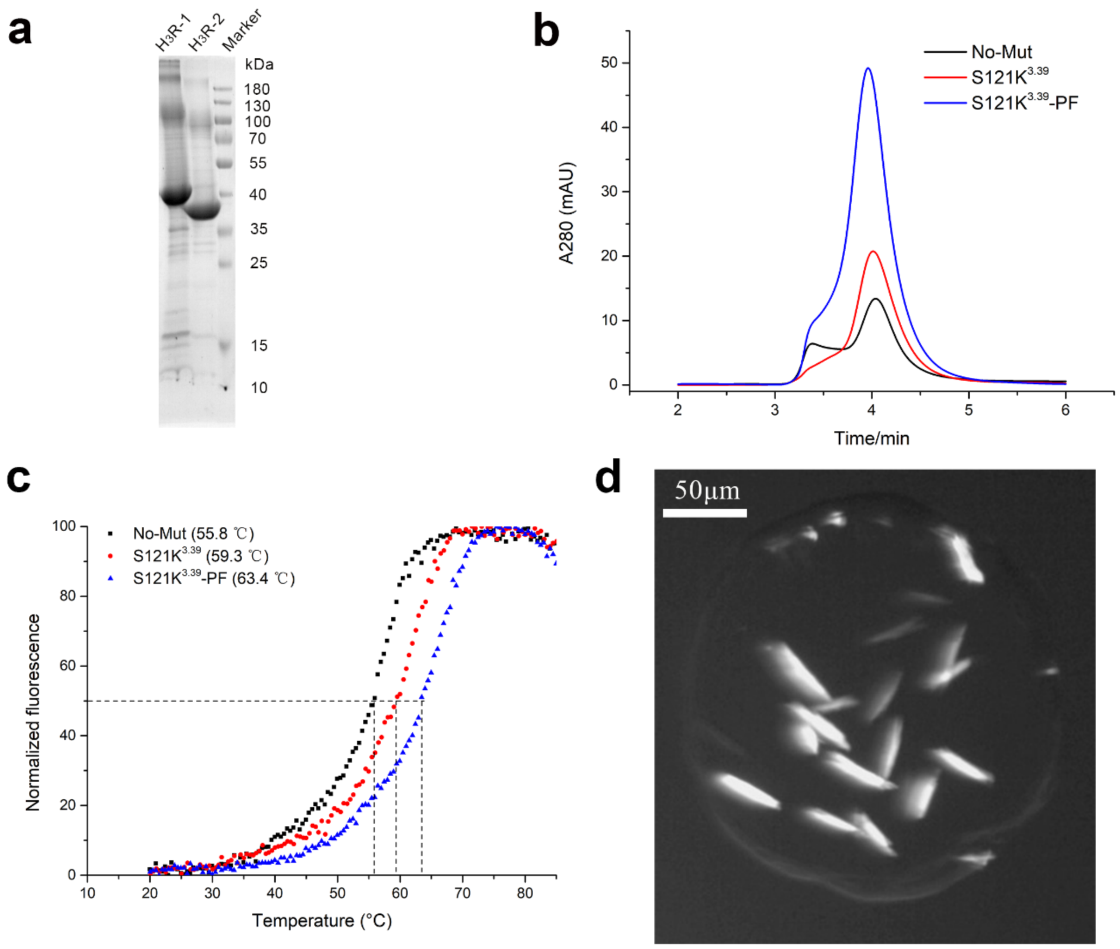

Supplementary Figure. 1. Protein purification and crystallization.

(a) SDS-PAGE of the purified H3R proteins in different steps. H3R-1 is the proteins eluted from the TALON IMAC resin, and H3R-2 is the final sample after tags removal by TEV protease digestion. Purification trials have been repeated independently at least three times with similar results. (**b**) Size exclusion chromatography results of the purified H3R proteins. No-Mut, the crystallized construct with the N/C-terminal/ICL3 truncated and BRIL inserted at the N-terminus and without the mutation and ligand (black). S121K$^{3.39}$, the crystallized construct with S121K$^{3.39}$ (red). S121K$^{3.39}$-PF, the crystallized construct with S121K$^{3.39}$ and PF-03654746 (blue). **(c)** Thermostability assays of the purified H3R proteins. Colors were presented as same as (**b**). (**d**) Crystals of H3R-PF-03654746 grown in LCP. Source data are provided as a Source Data File.

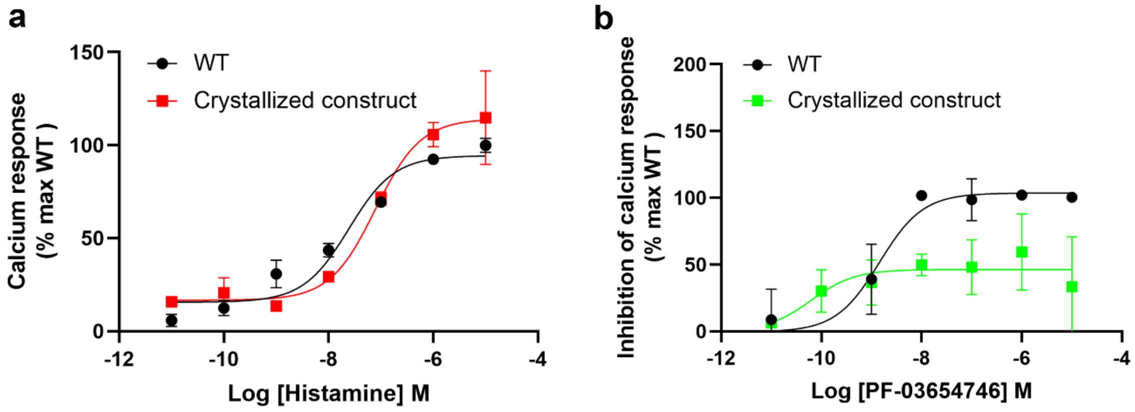

Supplementary Fig. 2. Functional validation of H_3R crystallized construct.

(**a**) Histamine-induced calcium mobilization of H_3R. (**b**) Inhibition of histamine-induced calcium mobilization of H_3R by PF-03654746. Data are shown as mean ± SEM, n = 3 independent replicates. WT represents wild-type. Source data are provided as a Source Data File.

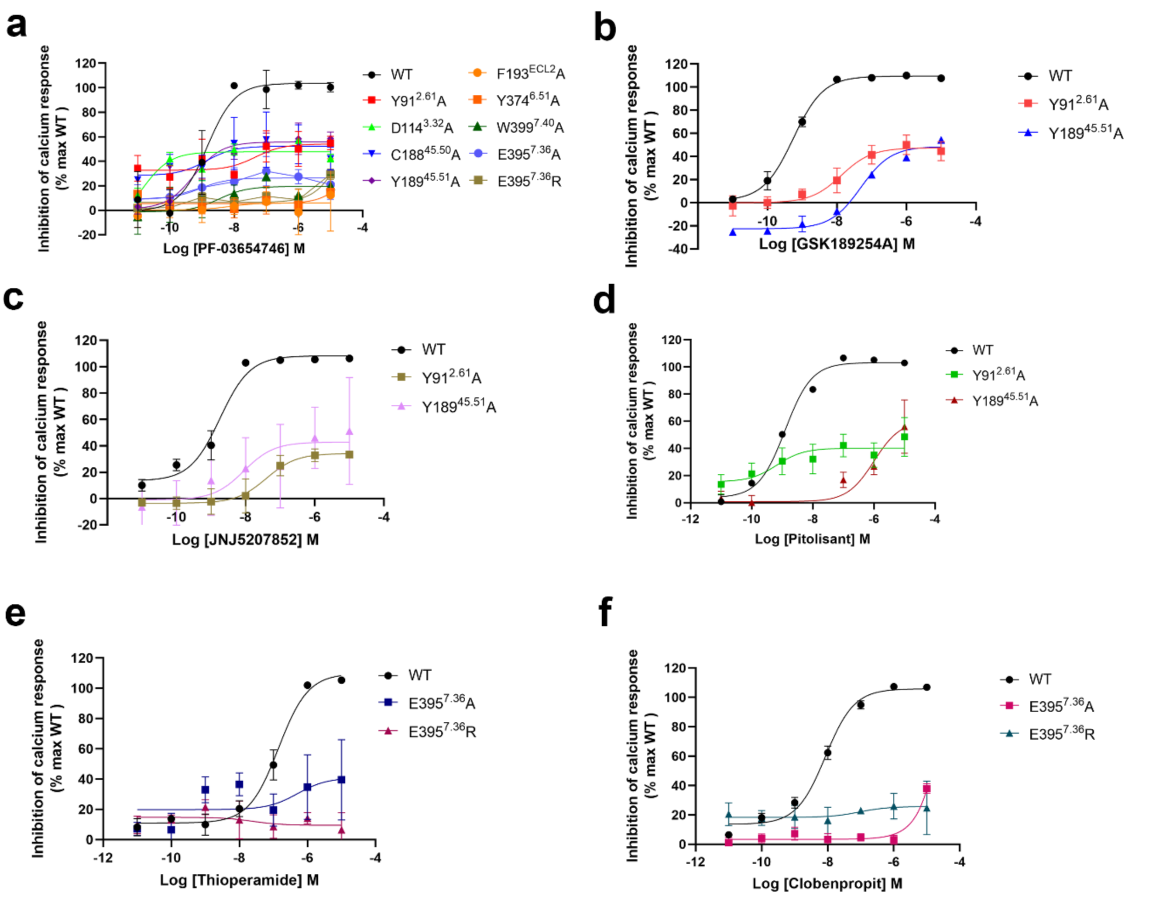

Supplementary Fig. 3. Inhibition of histamine-induced calcium mobilization for H_3R mutants by different antihistamines.

a-f, Results are presented as mean % of inhibition of calcium responses ± SEM, n = 3 independent replicates. WT represents wild-type. Source data are provided as a Source Data File.

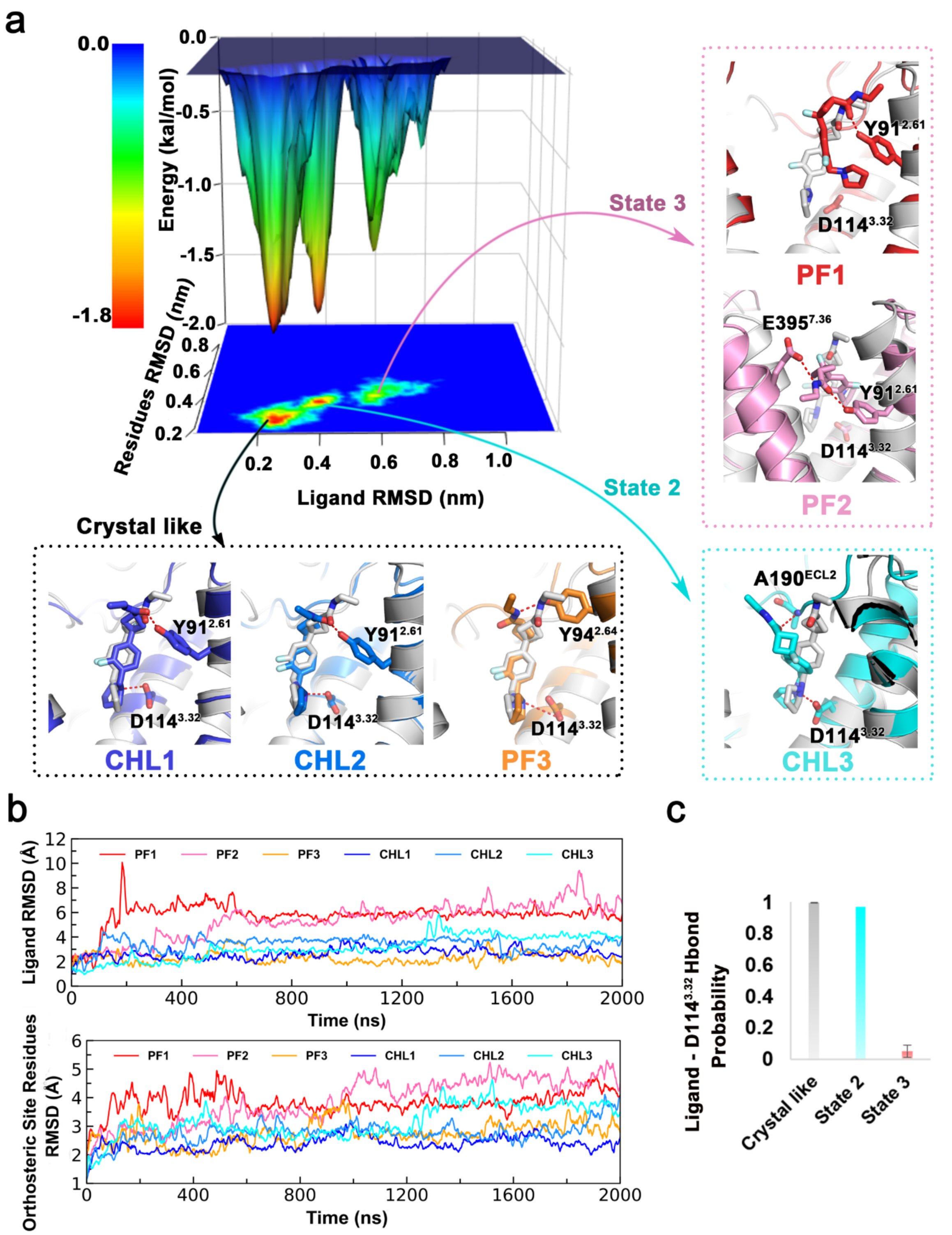

Supplementary Fig. 4. H_3R-PF-03654746 complex adopted three binding states in the presence or absence of cholesterol.

(a) Top, free energy landscape of H_3R-PF-03654746 complex generated by the last 500 ns trajectories in six simulations. CHL indicated H_3R/PF-03654746/cholesterol and PF indicated H_3R/PF-03654746. Two coordinates were defined according to the RMSD of PF-03654746 and that of orthosteric site residues as shown in panel **b**. Bottom, superimposition

of crystal complex (white) and representative conformations in crystal-like state extracted from simulations CHL1 (blue), CHL2 (dodger blue) and PF3 (orange) respectively. Right, superimposition of crystal complex (white) and typical conformations of state 2 and state 3. State 2 is generated by simulation CHL3 (cyan) and state 3 by PF1 (red) and PF2 (pink). PF-03654746 and residues involved in major interactions are depicted as sticks. Polar interactions are represented as red dash lines. (**b)** The RMSD values of ligand and orthosteric site residues during the simulation. The crystal conformation is set as the reference structure and coloring scheme is in line with panel a. (**c)** Occupancy of the salt bridge between ligand and $D114^{3.32}$ in three states. Values were calculated from the last 300 ns in each trajectory. Data were generated and graphed as mean ± SD for crystal-like state and state 3, but only as mean for state2. In crystal-like state, the error bar reflected the standard deviation among n=3 independent simulations and its value was 0.002. In state3, the standard deviation of n=2 independent simulations were 0.040. As only one simulation fell into state2, there was no error bar for this state.

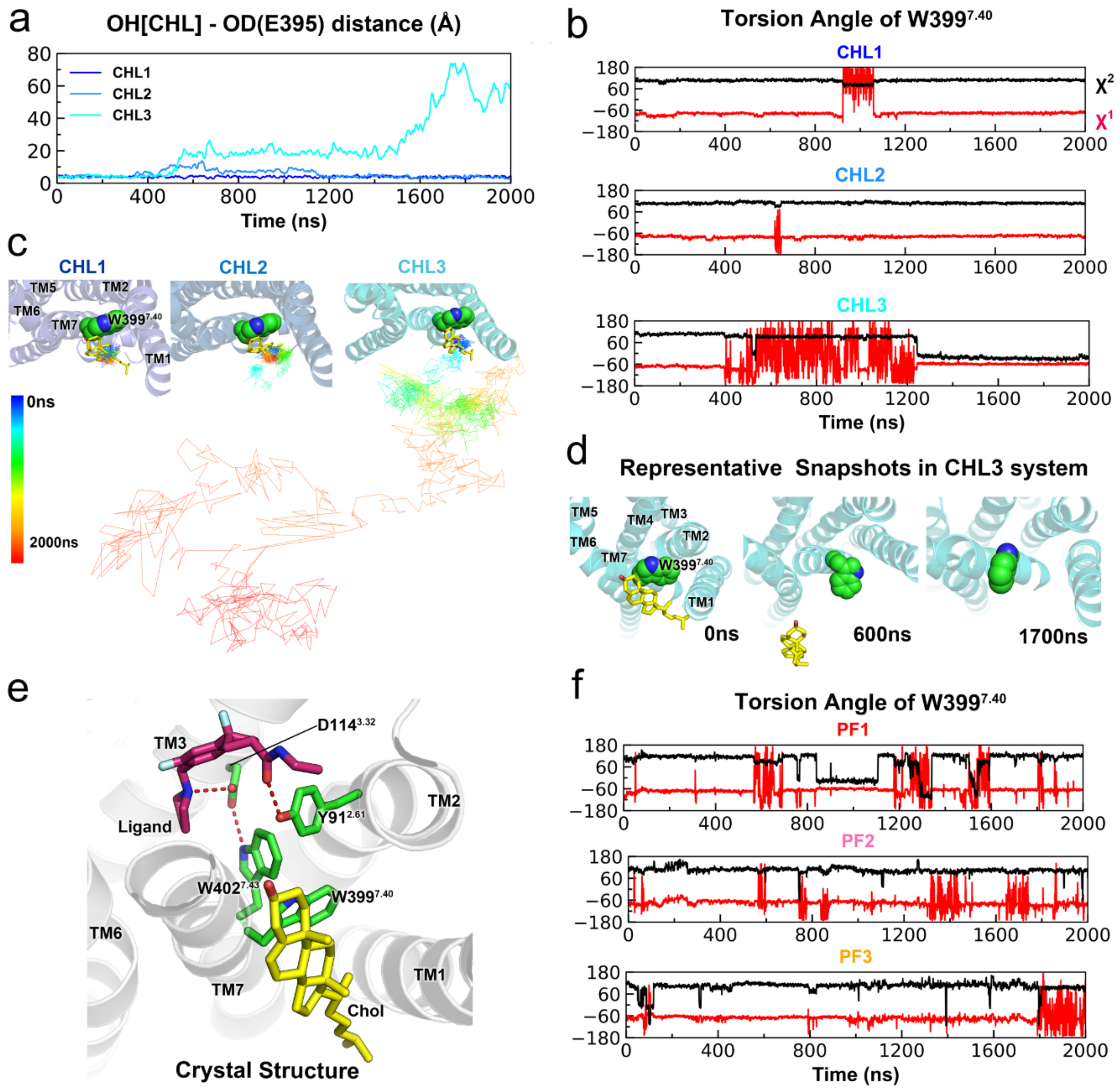

Supplementary Fig. 5. The impact of W399$^{7.40}$ on cholesterol binding and ligand-H$_3$R interactions.

(a) Time dependences of the distance between OH atom of cholesterol and OD atom of E395$^{7.36}$. (**b**) Time evolution of χ1 (red), χ2 (black) torsion angles of W399$^{7.40}$ side chain in three simulations with cholesterol. (**c**) Trajectory of the COM (center of mass) of cholesterol in simulations CHL1, CHL2, and CHL3. (**d**) Typical snapshots showing the dissociation progress of cholesterol in CHL3 caused by the rotameric changes of the side chain of W399$^{7.40}$. (**e**) T-shape π-π stackings between W399$^{7.40}$ and W402$^{7.43}$, Y91$^{2.61}$, and their involvement in ligand binding. (**f**) Time evolution of χ1 (red), χ2 (black) torsion angles of W399$^{7.40}$ side chain in three simulations without cholesterol.

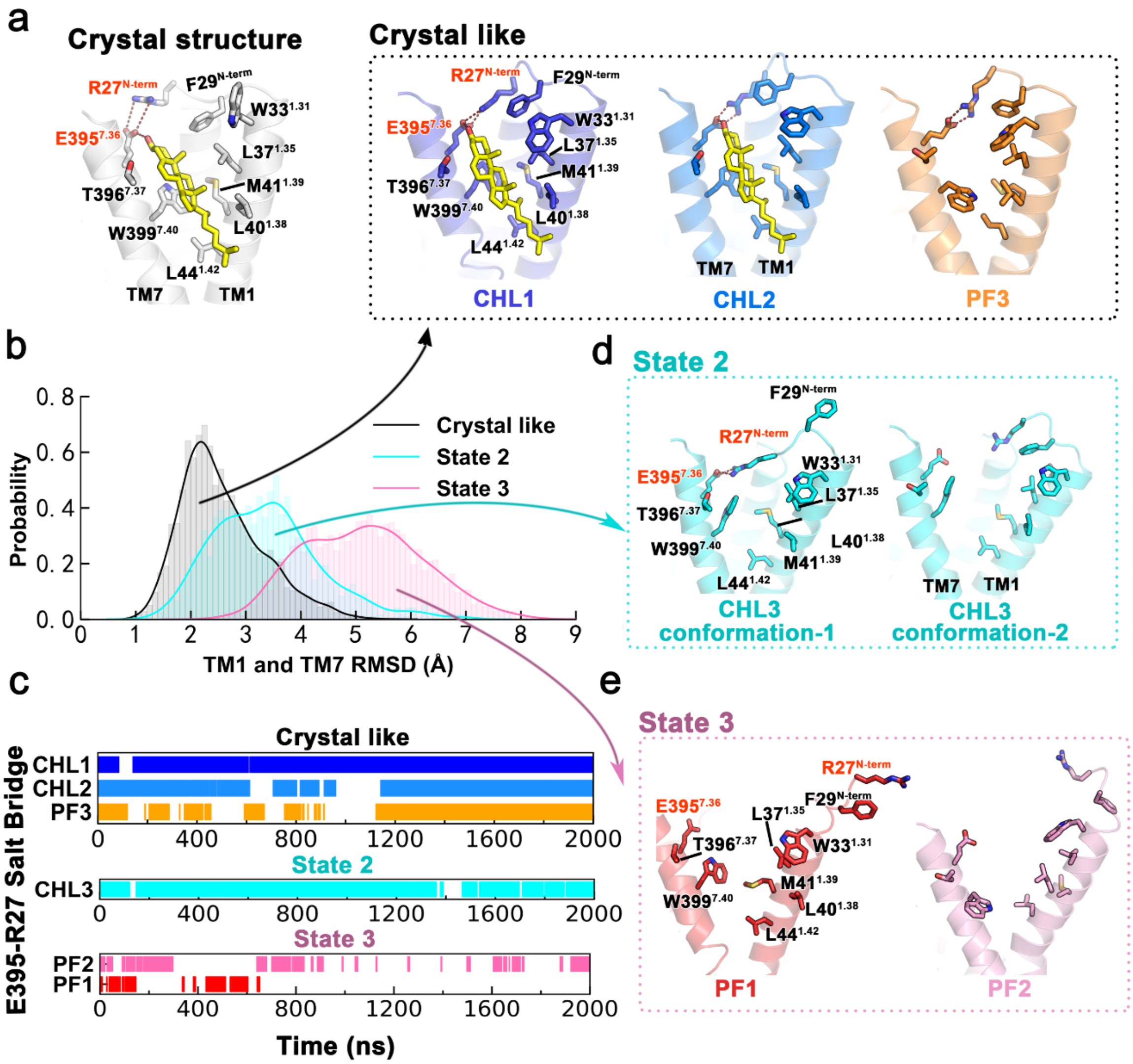

Supplementary Fig. 6. Cholesterol facilitated rearrangements of TM1-TM7 interface and stabilized a polar network of cholesterol-E395$^{7.36}$-R27$^{N\text{-term}}$.

Representative conformations of TM1-TM7 interface in MD simulations were shown according to the three complex states observed in Supplementary Fig. 2: crystal-like state (**a**), state 2 (**d**), state 3 (**e**). Residues involved in interactions were shown in sticks. Polar contacts were depicted as red dash lines. (**b**) Distribution of the RMSD of TM1 and TM7 in three complex states. Crystal-like state adopted the most stable TM1-TM7 conformation, while state 2 and state 3 were unstable. (**c**) Existence of E395$^{7.36}$-R27$^{N\text{-term}}$ interaction versus the simulation time in each trajectory.

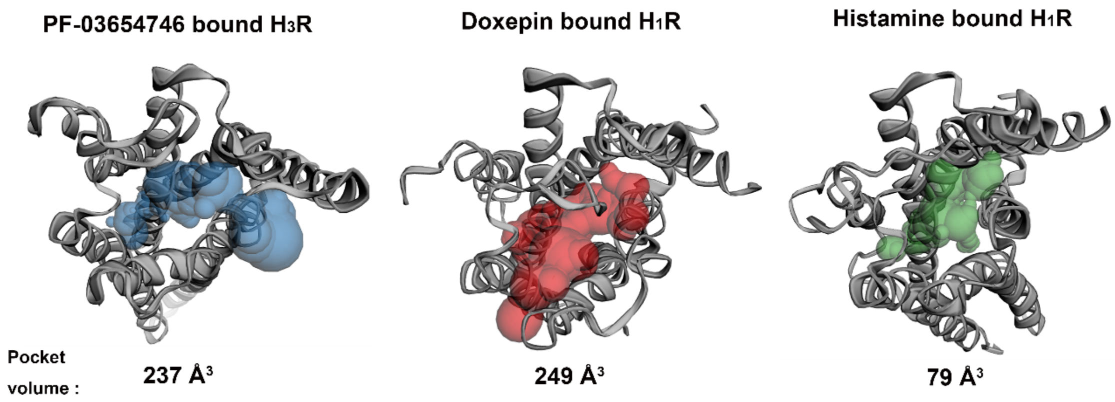

Supplementary Fig. 7. A comparison of the size of ligand-binding pocket among the antagonist PF-03654746-bound H_3R, antagonist doxepin-bound H_1R (PDB ID: 3RZE), and agonist histamine-bound H_1R (PDB ID: 7DFL). The size of the ligand binding pocket was calculated by CASTp 3.0 server.

Supplementary Table 1. Activities of different antihistamines for the wild-type and mutated H_3R in the calcium mobilization assays.

EC_{50}, IC_{50}, and E_{max} estimates represent the average and standard error of mean (SEM) from n = 3 independent experiments. E_{max} is defined as percentage of maximum response of wild-type (WT). N.D. represents no detectable activity as equilibrium could not be achieved at maximum agonist concentration for a reliable curve fitting.

H_3R	Histamine		PF-03654746	
	EC_{50} (nM)	E_{max} (%)	IC_{50} (nM)	E_{max} (%)
**WT**	24.96±9.99	100±3.82	1.45±0.51	100±3.75
**Crystal**	84.81±49.86	114.8±25.11	0.08±0.04	33.46±37.27
**W399A**	117.70±24.26	139.91±7.15	N.D.	19.67±9.24
**D114A**	162.67±17.19	121.27±11.52	1.94±1.03	36.13±10.08
**C188A**	99.60±27.40	141.29±12.28	1.09±0.44	42.15±9.37
**F193A**	26.35±10.95	145.33±5.03	N.D.	12.49±29.57
**Y374A**	53.60±23.52	134.82±2.81	N.D.	14.78±2.45
**Y91A**	14.04±64.82	154.10±0.21	66.80±46.09	53.26±4.78
**Y189A**	10.89±0.09	143.40±3.18	0.58±0.19	58.55±5.33
**E395A**	37.82±16.47	142.10±8.41	N.D.	20.99±11.98
**E395R**	45.82±31.95	111.46±24.04	N.D.	29.45±4.14

Supplementary Table 2. Calcium mobilization assays of the wild-type and mutated H_3R for docking results.

IC_{50} and E_{max} estimates represent the average and standard error of mean (SEM) from n = 3 independent experiments. E_{max} is defined as percentage of maximum response of wild-type (WT). N.D. represents no detectable activity as equilibrium could not be achieved at maximum agonist concentration for a reliable curve fitting. N.A. represents not available.

H_3R	JNJ5207852		GSK-189254A		Pitolisant		Thioperamide		Clobenpropit	
	IC_{50} (nM)	E_{max} (%)	IC_{50} (nM)	E_{max} (%)	IC_{50} (nM)	E_{max} (%)	IC_{50} (nM)	E_{max} (%)	IC_{50} (nM)	E_{max} (%)
**WT**	1.84±0.65	106.33±1.36	0.55±0.11	107.6±0.97	1.25±0.07	102±1.08	145.81±51.38	105.3±1.73	8.64±1.73	107.28±2.16
**Y91A**	42.70±26.63	33.25±1.61	14.17±9.70	44.59±8.22	N.D.	48.46±14.28	N.A.	N.A.	N.A.	N.A.
**Y189A**	1.23±0.95	51.18±40.69	48.54±7.16	54.36±2.98	N.D.	56.04±19.56	N.A.	N.A.	N.A.	N.A.
**E395A**	N.A.	N.A.	N.A.	N.A.	N.A.	N.A.	N.D.	39.59±26.52	N.D.	35.33±3.38
**E395R**	N.A.	N.A.	N.A.	N.A.	N.A.	N.A.	N.D.	6.43±11.36	N.D.	21.56±19.04

Supplementary Table 3. Crystallographic data collection and refinement statistics.

	**H_3R-PF-03654746**
**PDB ID**	**7F61**
**Data collection**	
Space group	$P\ 2_1\ 2_1\ 2$
Cell dimensions	
a, b, c (Å)	71.12, 165.62, 42.65
α, β, γ (°)	90, 90, 90
Resolution (Å)	29.90-2.60 (2.69-2.60)
R_{merge}	0.146 (0.714)
R_{pim}	0.117 (0.640)
$I/\sigma I$	6.79 (1.25)
CC	0.995 (0.828)
Completeness (%)	98.5 (97.5)
Redundancy	5.2 (4.5)
**Refinement**	
Resolution (Å)	29.90-2.60 (2.69-2.60)
No. reflections	15,979 (1564)
R_{work} / R_{free}	0.240/0.281 (0.308/0.297)
No. atoms	3,313
Average *B*-factors ($Å^2$)	45.3
RMSD	
Bond lengths (Å)	0.010
Bond angles (°)	1.66
Ramachandran plot (%)	
Favored	99.01
Allowed	0.99
Disallowed	0

#Data from one crystal were used to solve the structure.

*Highest resolution shell was shown in parenthesis.

Supplementary Table 4. The docking results of 10 ligands.

The unit of the docking score was kcal/mol. 10 ligands were ranked according to their K_i values from low to high. The K_i values were obtained from reported assays[1, 2, 3, 4].

Ligands	Docking score	K_i (nM)
Pitolisant	-8.46	0.16
GSK189254A	-10.90	0.2
LML134	-13.14	0.3
Clobenpropit	-10.91	0.4
JNJ5207852	-11.03	0.6
GSK334429	-11.65	0.8
MK-0249	-10.73	1.7
PF-03654746	-10.59	3.2
Bavisant	-10.33	5.4
Thioperamide	-9.84	31

Supplementary Table 5. Residues within 4 Å from PF-03654746 in H$_3$R and their equivalents in the related aminergic receptors.

H$_3$R	H$_1$R	H$_2$R	H$_4$R	α$_{2A}$AR	α$_{2C}$AR	M$_1$R
Y91$^{2.61}$	N84$^{2.61}$	S75$^{2.61}$	Y72$^{2.61}$	S105$^{2.61}$	S108$^{2.61}$	Y82$^{2.61}$
Y94$^{2.64}$	Y87$^{2.64}$	Y78$^{2.64}$	H75$^{2.64}$	N108$^{2.64}$	N111$^{2.64}$	Y85$^{2.64}$
V95$^{2.65}$	L88$^{2.65}$	Q79$^{2.65}$	T76$^{2.65}$	E109$^{2.65}$	E112$^{2.65}$	L86$^{2.65}$
W110$^{3.28}$	W103$^{3.28}$	Y94$^{3.28}$	W90$^{3.28}$	Y124$^{3.28}$	Y127$^{3.28}$	W101$^{3.28}$
L111$^{3.29}$	L104$^{3.29}$	T95$^{3.29}$	L91$^{3.29}$	L125$^{3.29}$	L128$^{3.29}$	L102$^{3.29}$
D114$^{3.32}$	D107$^{3.32}$	D98$^{3.32}$	D94$^{3.32}$	D128$^{3.32}$	D131$^{3.32}$	D105$^{.32}$
Y115$^{3.33}$	Y108$^{3.33}$	V99$^{3.33}$	Y95$^{3.33}$	V129$^{3.33}$	V132$^{3.33}$	Y106$^{3.33}$
C118$^{3.36}$	S111$^{3.36}$	C102$^{3.36}$	C98$^{3.36}$	C132$^{3.36}$	C135$^{3.36}$	S109$^{3.36}$
C188$^{45.50}$	C180$^{45.50}$	C174$^{45.50}$	C164$^{45.50}$	C203$^{45.50}$	C202$^{45.50}$	C178$^{45.50}$
Y189$^{45.51}$	E181$^{45.51}$	K175$^{45.51}$	E165$^{45.51}$	E204$^{45.51}$	G203$^{45.51}$	Y179$^{45.51}$
F193^{ECL2}	Y185^{ECL2}	Q179^{ECL2}	F169^{ECL2}	N206^{ECL2}	N205^{ECL2}	L183^{ECL2}
Y374$^{6.51}$	Y431$^{6.51}$	Y250$^{6.51}$	Y319$^{6.51}$	F405$^{6.51}$	F398$^{6.51}$	Y381$^{6.51}$
Y394$^{7.35}$	H450$^{7.35}$	E270$^{7.35}$	Y340$^{7.35}$	F423$^{7.35}$	F419$^{7.35}$	W400$^{7.35}$
E395$^{7.36}$	M451$^{7.36}$	A271$^{7.36}$	R341$^{7.36}$	K424$^{7.36}$	K420$^{7.36}$	E401$^{7.36}$
F398$^{7.39}$	I454$^{7.39}$	L274$^{7.39}$	F344$^{7.39}$	F427$^{7.39}$	F423$^{7.39}$	Y404$^{7.39}$
W399$^{7.40}$	W455$^{7.40}$	W275$^{7.40}$	W345$^{7.40}$	W428$^{7.40}$	W424$^{7.40}$	W405$^{7.40}$
L401$^{7.42}$	G457$^{7.42}$	G277$^{7.42}$	Q347$^{7.42}$	G430$^{7.42}$	G426$^{7.42}$	C407$^{7.42}$
W402$^{7.43}$	Y458$^{7.43}$	Y278$^{7.43}$	W348$^{7.43}$	Y431$^{7.43}$	Y427$^{7.43}$	Y408$^{7.43}$

[1]The alignment were sequence-based for the related aminergic receptors

[2]Conserved residues are shown in green.

Supplementary References

1. Panula P*, et al.* International Union of Basic and Clinical Pharmacology. XCVIII. Histamine Receptors. *Pharmacol Rev* **67**, 601-655 (2015).
2. Nieto-Alamilla G, Márquez-Gómez R, García-Gálvez AM, Morales-Figueroa GE, Arias-Montaño JA. The Histamine H3 Receptor: Structure, Pharmacology, and Function. *Mol Pharmacol* **90**, 649-673 (2016).
3. Troxler T*, et al.* The Discovery of LML134, a Histamine H3 Receptor Inverse Agonist for the Clinical Treatment of Excessive Sleep Disorders. *ChemMedChem* **14**, 1238-1247 (2019).
4. Leurs R, Vischer HF, Wijtmans M, de Esch IJ. En route to new blockbuster anti-histamines: surveying the offspring of the expanding histamine receptor family. *Trends Pharmacol Sci* **32**, 250-257 (2011).
