# Supplementary material for: Structural basis for recognition of antihistamine drug by human histamine receptor
Source: Nat Commun. 2022 Oct 15;13:6105. doi: 10.1038/s41467-022-33880-y (PMC9569329; doi:10.1038/s41467-022-33880-y)
Supplement: Supplementary file 3 — Reporting Summary [file 41467_2022_33880_MOESM3_ESM.pdf]

nature portfolio

Corresponding author(s): Haitao Zhang

Last updated by author(s): Sep 5, 2022

Reporting Summary

Nature Portfolio wishes to improve the reproducibility of the work that we publish. This form provides structure for consistency and transparency in reporting. For further information on Nature Portfolio policies, see our Editorial Policies and the Editorial Policy Checklist.

Statistics

For all statistical analyses, confirm that the following items are present in the figure legend, table legend, main text, or Methods section.

n/a	Confirmed	
[ ]	[x]	The exact sample size (*n*) for each experimental group/condition, given as a discrete number and unit of measurement
[ ]	[x]	A statement on whether measurements were taken from distinct samples or whether the same sample was measured repeatedly
[x]	[ ]	The statistical test(s) used AND whether they are one- or two-sided<br>*Only common tests should be described solely by name; describe more complex techniques in the Methods section.*
[x]	[ ]	A description of all covariates tested
[x]	[ ]	A description of any assumptions or corrections, such as tests of normality and adjustment for multiple comparisons
[ ]	[x]	A full description of the statistical parameters including central tendency (e.g. means) or other basic estimates (e.g. regression coefficient) AND variation (e.g. standard deviation) or associated estimates of uncertainty (e.g. confidence intervals)
[x]	[ ]	For null hypothesis testing, the test statistic (e.g. *F*, *t*, *r*) with confidence intervals, effect sizes, degrees of freedom and *P* value noted<br>*Give P values as exact values whenever suitable.*
[x]	[ ]	For Bayesian analysis, information on the choice of priors and Markov chain Monte Carlo settings
[x]	[ ]	For hierarchical and complex designs, identification of the appropriate level for tests and full reporting of outcomes
[x]	[ ]	Estimates of effect sizes (e.g. Cohen's *d*, Pearson's *r*), indicating how they were calculated

Our web collection on statistics for biologists contains articles on many of the points above.

Software and code

Policy information about availability of computer code

Data collection	The X-ray diffraction data of crystals were collected at the BL18U1 beamline of Shanghai Synchrotron Radiation Facility.
Data analysis	The following softwares were used in this study: HKL3000, COOT 0.9.6, Phenix 1.19.2, Pymol 2.0, GraphPad Prism 9.3.1, GROMACS 2020.

For manuscripts utilizing custom algorithms or software that are central to the research but not yet described in published literature, software must be made available to editors and reviewers. We strongly encourage code deposition in a community repository (e.g. GitHub). See the Nature Portfolio guidelines for submitting code & software for further information.

Data

Policy information about availability of data

All manuscripts must include a data availability statement. This statement should provide the following information, where applicable:

- Accession codes, unique identifiers, or web links for publicly available datasets
- A description of any restrictions on data availability
- For clinical datasets or third party data, please ensure that the statement adheres to our policy

The structural data generated in this study have been deposited in the Protein Data Bank (http://www.pdb.org/) under accession code 7F61. The other data generated in this study are provided in the Supplementary Information and Source Data file. Source data are provided with this paper.

Field-specific reporting

Please select the one below that is the best fit for your research. If you are not sure, read the appropriate sections before making your selection.

- [x] Life sciences
- [ ] Behavioural & social sciences
- [ ] Ecological, evolutionary & environmental sciences

For a reference copy of the document with all sections, see nature.com/documents/nr-reporting-summary-flat.pdf

Life sciences study design

All studies must disclose on these points even when the disclosure is negative.

Sample size	No statistical methods were used to predetermine sample size, the size of crystals sample in lipidic cubic phase is determined by protein stability and overlay precipitant conditions. X-ray diffraction data from one best-diffracting crystal were allowed us to integrate a 98.5% complete data and used to solve the structure. Calcium mobilization assays were performed as 3 independent experiments (n=3). Molecular dynamics simulations were replicated to perform three independent simulations and each of the three simulations was run up to 2-µs.
Data exclusions	No data were excluded from the analyses.
Replication	Calcium mobilization assays in this study have been repeated by three independent experiments. Crystallization trials have been successfully repeated at least four times with reproducibly diffracting crystals.
Randomization	Randomization was not relevant to this study. This was not an animal study or clinical trial in which randomization would be important. The experiments in this study were validated by sequencing, SDS-PAGE and X-ray structure determination.
Blinding	No Blinding was required for this study since no animals or human subjects were involved.

Reporting for specific materials, systems and methods

We require information from authors about some types of materials, experimental systems and methods used in many studies. Here, indicate whether each material, system or method listed is relevant to your study. If you are not sure if a list item applies to your research, read the appropriate section before selecting a response.

Materials & experimental systems

n/a	Involved in the study	
x		Antibodies
	x	Eukaryotic cell lines
x		Palaeontology and archaeology
x		Animals and other organisms
x		Human research participants
x		Clinical data
x		Dual use research of concern

Methods

n/a	Involved in the study	
x		ChIP-seq
x		Flow cytometry
x		MRI-based neuroimaging

Eukaryotic cell lines

Policy information about cell lines

Cell line source(s)	sf9 (Gibco, cat#12659017)
Authentication	Used as expression stains only, independent verification after purchase is not required.
Mycoplasma contamination	Cell lines were free from mycoplasma contamination.
Commonly misidentified lines (See ICLAC register)	No commonly misidentified cell lines were used.
